# Development and Validation of a Toxoplasma Infection-Associated Risk Model for Prognostic Stratification and Treatment Guidance in Glioma

**DOI:** 10.3390/biology15080633

**Published:** 2026-04-17

**Authors:** Le Pan, Qian Hu, Qili Yu, Xueyu Zhang, Yangfei Chen, Fei Chen, Weidong Deng

**Affiliations:** 1 Yunnan Provincial Key Laboratory of Animal Nutrition and Feed, Faculty of Animal Science and Technology, Yunnan Agricultural University, Kunming 650201, China; pl6677321@163.com; 2 Hangzhou Xiaoshan Donghai Aquaculture Co., Ltd., Hangzhou 311200, China; yqlwf09194129@163.com (Q.Y.); zxy793856@163.com (X.Z.); cyf343536@163.com (Y.C.); 3 Center for Medical Genetics, Hunan Key Laboratory of Medical Genetics, School of Life Sciences, Central South University, 110 Xiangya Road, Changsha 410078, China; huqian@sklmg.edu.cn

**Keywords:** *Toxoplasma gondii*, glioma, interaction, cross-kingdom regulation

## Abstract

Gliomas are aggressive brain tumors with poor prognosis. The contribution of *Toxoplasma gondii (T. gondii)*-related transcriptional programs to glioma remains unclear. We identified *T. gondii* infection-related genes from neuroepithelial cell transcriptomes, mapped them to TCGA and CGGA glioma datasets, and validated their expression via RT-qPCR. A prognostic signature (TGRisk) was constructed via Cox and LASSO regression and validated across independent cohorts. Functional, immune, and drug sensitivity analyses were conducted. Forty infection-related genes were identified, enriched in stress responses, microRNA regulation, ribosome biogenesis, and metabolism. The 13-gene TGRisk model significantly separated survival between high- and low-risk groups. A nomogram combining TGRisk with clinical features improved prediction accuracy. High-risk tumors showed immune activation and higher infiltration of CD8^+^ T cells, Tregs, macrophages, and neutrophils, while low-risk tumors showed enhanced neuronal signaling and NK cell activity. Drug sensitivity prediction suggested low-risk patients were more responsive to temozolomide and bortezomib, whereas high-risk patients were more sensitive to dasatinib and ruxolitinib. We developed a novel *T. gongdii* infection-related gene signature that stratifies glioma patients by prognosis, immune features, and therapeutic vulnerabilities. These findings suggest host–*T. gondii* interactions and a potential biomarker for patient stratification and personalized therapy.

## Simple Summary

*Toxoplasma gondii* is a common parasite linked to brain tumors. Evidence has linked its infection to glioma risk and progression, but whether infection-related gene expression patterns are clinically useful prognostic markers is unclear. We identified genes linked to *T. gondii* infection from neuroepithelial cell data and validated their expression via RT-qPCR. These genes were compared against glioma datasets. A risk prediction model was constructed and validated across independent cohorts. Pathway, immune cell, and drug response analyses were conducted to assess biological and clinical significance. Forty infection-related genes were identified. The 13-gene TGRisk model separated patients into high- and low-risk groups with significantly different survival times. A prediction chart combining TGRisk with clinical features further improved prediction accuracy. High-risk tumors showed immune-related signs, while low-risk tumors showed nerve-related signaling and higher natural killer cell activity. Drug response analysis suggested potential treatment options for each group. In this research, we identified a new gene signature linked to *T. gondii* infection that effectively classifies glioma patients according to expected outcomes, immune features, and potential treatment responses. These findings point to potential link between the host and *T. gondii*, and suggest a potential tool for patient classification and personalized therapy.

## 1. Introduction

Gliomas are the most common and aggressive primary tumors of the central nervous system, of which glioblastoma (GBM) is the most lethal subtype. Despite advances in surgery, radiotherapy, and chemotherapy, the overall prognosis of glioma patients remains poor, with median survival for GBM rarely exceeding 15 months [1,2,3]. Accumulating evidence has highlighted the importance of the tumor microenvironment, immune dysregulation, and genetic heterogeneity in shaping glioma progression and therapeutic resistance [4,5,6]. Therefore, the identification of reliable molecular biomarkers is critical for improving prognosis prediction and guiding individualized therapy [7,8].

In parallel, infectious agents have been increasingly recognized for their role in modulating cancer biology [9,10]. Among them, *Toxoplasma gondii* (*T. gondii*), an obligate intracellular protozoan parasite, has attracted attention due to its ability to invade neural tissue and manipulate host immune and metabolic pathways [11,12]. Epidemiological evidence consistently links *T. gondii* infection to an increased risk of brain tumors. This association is underscored by a recent meta-analysis of seven observational studies, which reported a pooled odds ratio of 1.96 for *T. gondii* seropositivity in relation to overall brain tumor risk. Notably, subgroup analyses specifically identified a significant positive correlation between T. gondii infection and glioma [13]. Further supporting this link, a 2024 case–control study from Egypt revealed a strong association between *T. gondii* seropositivity and pediatric brain tumors [14]. Beyond epidemiological correlations, molecular studies have begun to elucidate potential mechanisms by which *T. gondii* may directly influence glioma biology.

Rather than considering infection as a general source of shared pathways, we focus on a parasite with strong neurotropism that can establish long-term latency in the brain, and which may influence glioma development by shaping the local immune environment in which GBM evolves. Experimental evidence has shown that *T. gondii* infection can directly or indirectly alter the glioma microenvironment and affect glioma cell biology. Mechanistically, the parasite virulence factor ROP18 inhibits apoptosis in glioma cells by targeting the host P2X1 receptor through the mitochondrial pathway [15]. *T. gondii*-infected microglial cells release exosomes containing miR-21, which are internalized by glioma cells and downregulate the expression of tumor suppressor genes PTEN, FoxO1, and PDCD4, thereby promoting glioma cell proliferation [16]. Moreover, *T. gondii* infection induces T cell infiltration into brain tumors and reprograms myeloid cell phenotypes, converting an immunosuppressive microenvironment into a T cell-supportive state [17]. Previous studies have shown that *T. gondii* infection can alter host gene expression, immune responses, and apoptosis regulation, processes that overlap with mechanisms of glioma pathogenesis [18,19]. Together, these findings reveal a complex bidirectional interaction between *T. gondii* infection and glioma, providing a biological basis for exploring the prognostic value of infection-related genes in glioma. These observations motivate a specific mechanistic hypothesis: a *T. gondii*-associated transcriptional signature may capture a distinct, chronically remodeled immune-metabolic state (including immune surveillance pressure, antigen presentation remodeling, and cell-death/stress adaptation) that is relevant to glioma aggressiveness and therapy resistance, rather than merely recapitulating broad inflammatory pathway activation. If this hypothesis is true, such a parasite-linked signature should provide prognostic and microenvironmental information that is not fully explained by conventional pathway enrichment or generic immune signatures.

Recent advances in bioinformatics and the availability of large-scale transcriptomic datasets have now made it possible to systematically identify infection-related gene signatures and integrate them into prognostic models [20,21]. Building on this rationale, we leveraged bioinformatics analysis to examine the expression and clinical significance of *T. gondii* infection-related genes in glioma. We aimed to construct and validate a prognostic risk model and to determine whether the parasite-related signature offers unique prognostic stratification and interpretable immune/therapeutic associations beyond generic pathway analysis [22].

## 2. Materials and Methods

### 2.1. Data Download and Preprocessing

The microarray dataset was obtained from the Gene Expression Omnibus (GEO) database (GSE22986), which analyzed human neuroepithelioma SK-N-MC cells 20 h after *T. gondii* infection. The arrays were generated on the Affymetrix Human Exon 1.0 ST platform (GPL5175, transcript [gene] version) [23]. In this study, we focused on both the 2F and CTG arms. Probe/transcript cluster identifiers were annotated to HGNC gene symbols using the annotation field from GPL5175. When multiple gene symbols were listed, only the first was retained. For genes represented by multiple probe sets, the average expression value was calculated to obtain a single gene-level value. This preprocessing resulted in a gene × sample expression matrix of log-transformed intensity values for downstream analysis.

### 2.2. Differential Expression Analysis

Differential expression (DE) analysis was performed at two levels. For each strain (2F and CTG), paired comparisons between infected samples and their matched controls from the same replicate were conducted using the limma package with empirical Bayes moderation. *p* values were adjusted using the Benjamini–Hochberg method, and genes with |log2FC| > 0.585 and adjusted *p* < 0.05 were defined as differentially expressed genes (DEGs) (upregulated: log2FC > 0.585; downregulated: log2FC < −0.585). To identify cross-strain consensus signals, Robust Rank Aggregation (RRA) was applied separately to the ranked DE gene lists from 2F and CTG in both upregulated and downregulated directions, retaining genes with RRA-adjusted *p* < 0.05. In addition, a combined analysis was performed by merging 2F and CTG infected samples into an “infected” group and their matched controls into a “control” group, fitting a paired model and detecting DEGs under the same thresholds for infected versus control. The final infection-related gene set was defined as the intersection of RRA-significant genes with consistent directions and DEGs from the combined model.

### 2.3. Gene Ontology (GO) and Kyoto Encyclopedia of Genes and Genomes (KEGG) Analyses

To explore the potential biological functions of the *T. gondii*-related genes, GO and KEGG pathway enrichment analyses were carried out using the cluster Profiler R package version 4.0 [24]. The GO analysis included three domains: biological processes (BP), molecular functions (MF), and cellular components (CC), while the KEGG analysis was used to identify significantly enriched signaling pathways. Genes with adjusted *p* < 0.05 were considered statistically significant.

### 2.4. Glioma Data Collection and Preprocessing

Public databases provided transcriptomic profiles and clinical annotations of glioma patients. The training cohort consisted of TCGA-GBMLGG cases downloaded from the UCSC Xena platform, including overall survival time/status, age, sex, race, and histology. Two independent validation cohorts (CGGA311 and CGGA668) with matched clinical information were retrieved from the CGGA portal [25]. Expression data provided in FPKM format were transformed to TPM and further converted to log2 (TPM+1). Gene identifiers were harmonized across cohorts. Samples with zero follow-up time or incomplete survival information were excluded. To minimize cross-cohort effects without merging datasets, each cohort was processed independently, and analyses were restricted to the intersection of genes across cohorts. Notably, no tumor–normal differential expression was available for glioma; instead, we projected the predefined *T. gondii* infection-related gene set (derived from the parasite infection analysis) onto the glioma cohorts and directly employed these genes as candidate features.

### 2.5. Development of the Prognostic Model

Using the infection-related gene set as the feature space, a survival model was constructed and validated in the training cohort. First, univariate Cox proportional hazards regression was performed for each gene, and those with *p* < 0.05 were retained for further analysis. Second, feature selection was carried out using a LASSO-Cox regression model, with the optimal penalty parameter (λ) determined by 10-fold cross-validation at the minimum cross-validated partial likelihood deviance. Third, the selected genes were fitted into a multivariate Cox regression model to obtain the final coefficients. We stratified patients into high- and low-risk groups using the median risk score in the training set. Kaplan–Meier survival analysis with log-rank tests, calculated time-dependent ROC curves with their corresponding AUC values at 1, 3, and 5 years, as well as the concordance index (C-index).

### 2.6. Construction of the Prognostic Nomogram

A multivariable prognostic model was constructed in the TCGA-GBMLGG training cohort by integrating the infection-derived risk score with key clinicopathological variables (histological subtype, age, sex, and race). All variables were entered simultaneously into a Cox proportional hazards model. Prior to modeling, continuous predictors were z-standardized, and categorical predictors were treated as factors. Based on the linear predictors, a nomogram was generated to estimate overall survival (OS) probabilities at 1, 3, and 5 years. Model calibration was assessed using bootstrap-corrected calibration curves with 1000 resamples at each time point. Both apparent estimates and bias-corrected estimates were reported.

### 2.7. Gene Set Enrichment and Variation Analyses

To investigate pathways associated with infection-derived risk stratification, we compared high- and low-risk glioma groups using gene set enrichment analysis (GSEA) and gene set variation analysis (GSVA) based on KEGG and Gene Ontology (GO) collections (Biological Process, Cellular Component, and Molecular Function) annotated to HGNC symbols and restricted to genes expressed in each cohort [26,27].

For GSEA, genes were ranked by log2 fold change (high-risk vs. low-risk) estimated with limma. Analyses were performed with 1000 phenotype permutations. Pathways were considered significantly enriched when |normalized enrichment score (NES)| > 1, nominal *p* < 0.05, and FDR q < 0.25. Enrichment results were reported for both directions (upregulated in high-risk and upregulated in low-risk groups).

For GSVA, sample-level enrichment scores were calculated using the GSVA R package on log2 (TPM+1)-transformed expression matrices processed within each cohort. Pathway activity differences between risk groups were assessed by GSVA scores, with significance defined as FDR < 0.05 (Benjamini–Hochberg adjustment).

Tumor-infiltrating immune cell fractions were estimated using CIBERSORT with the LM22 leukocyte signature matrix and 1000 permutations [28]. For RNA-seq expression matrices (log2 [TPM+1]), quantile normalization was disabled, and samples with CIBERSORT deconvolution *p* ≥ 0.05 were excluded.

Immune functional activity was quantified using single-sample GSEA (ssGSEA) implemented in the GSVA package, based on curated immune-related GMT collections (HGNC-mapped gene symbols, duplicates averaged, genes with zero mean removed) [29]. To facilitate comparison across gene sets, ssGSEA scores were normalized by min–max scaling to the range: [0, 1]. We applied the Wilcoxon rank-sum test to compare immune cell proportions and immune function scores between high- and low-risk groups.

### 2.8. Clinicopathological and Immune Features

Clinicopathological characteristics (including histological subtype, age, sex, and race) were integrated with the risk score to examine their distribution across high- and low-risk groups. Age was dichotomized, with 65 years as the cutoff, and this threshold itself was excluded from statistical testing. Associations between risk groups and clinical features were evaluated using the chi-square test.

In addition, immune subtype information was obtained from a classification file based on immune modeling and matched to TCGA samples. Subtypes with very small sample sizes were excluded to ensure robust comparisons. The distribution of immune subtypes between high- and low-risk groups was compared using the chi-square test.

### 2.9. Drug Sensitivity Prediction

The potential chemotherapeutic response was assessed using the pRRophetic algorithm to predict the half-maximal inhibitory concentration (IC50) of candidate drugs [30]. The predicted IC50 values were then compared between the high- and low-risk groups.

### 2.10. RNA Extraction and Quantitative Real-Time PCR (RT-qPCR)

To validate the expression patterns of the 13 genes in the TGRisk model, RT-qPCR was performed using human primary astrocytes (HA) (FENGHUISHENGWU, Changsha, Hunan, China) as the normal control, U251 (FENGHUISHENGWU, Changsha, Hunan, China) as the low-risk glioma model, and T98G (FENGHUISHENGWU, Changsha, Hunan, China) as the high-risk, temozolomide-resistant model. Total RNA was extracted from cells using TRIzol reagent (Invitrogen, Carlsbad, CA, USA) and quantified with a NanoDrop 2000 spectrophotometer (Thermo Fisher Scientific, Waltham, MA, USA). Reverse transcription was conducted using the PrimeScript RT Master Mix (Takara, Kusatsu, Shiga, Japan) to synthesize cDNA from 1 ug of total RNA. Quantitative PCR was then performed on a StepOnePlus Real-Time PCR System (Applied Biosystems, Foster City, CA, USA) using SYBR Green Premix Ex Taq II (Takara, Kusatsu, Shiga, Japan) to detect the 13 genes (APH1B, MMD, HK2, ZNF217, RACGAP1, SAFB, CSRNP3, ZC3H6, VIPR2, CA13, DUSP5, ULBP1 and JUP). GAPDH was used as the internal control, and relative mRNA expression levels were calculated using the 2^−ΔΔCt^ method based on three independent biological replicates.

## 3. Results

### 3.1. Identification and Functional Enrichment Analysis of T. gondii Infection-Related Genes

**Figure 1:**
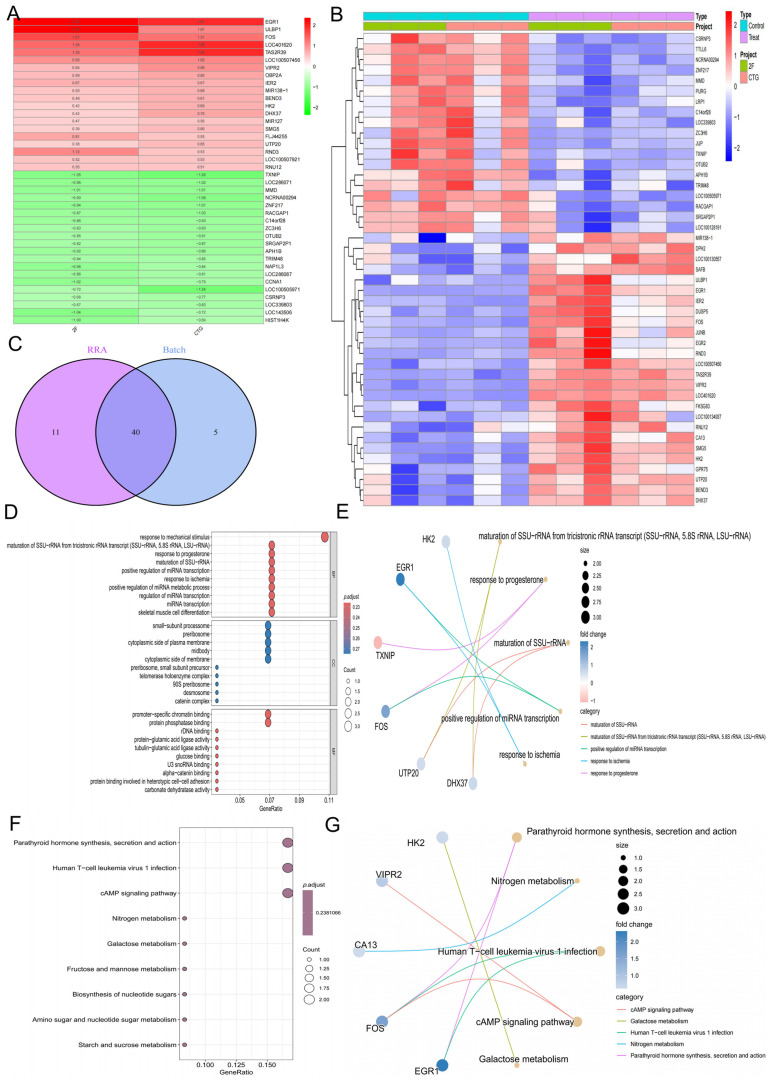
Identification and functional enrichment analysis of *T. gondii* infection-related genes. (**A**) Heatmap showing the top 20 upregulated and downregulated genes identified by the robust rank aggregation (RRA) algorithm (|logFC| > 0.585, adjusted *p* < 0.05). (**B**) Heatmap of differentially expressed genes (DEGs) from the pooled analysis of 2F- and CTG-infected samples versus matched controls. (**C**) Venn diagram depicting the overlap between RRA-derived DEGs and pooled DEGs, yielding 40 consistently significant *T. gondii* infection-related genes. (**D**,**E**) Gene Ontology (GO) enrichment analyses of the 40 genes, highlighting biological processes (BP), molecular functions (MF), and cellular components (CC), such as response to mechanical stimulus, regulation of microRNA transcription, ribosome biogenesis, and protein binding activities. (**F**,**G**) Kyoto Encyclopedia of Genes and Genomes (KEGG) pathway enrichment analyses showing significant associations with signaling and metabolic pathways, including cAMP signaling, nitrogen metabolism, and carbohydrate metabolism (galactose, fructose, mannose, and nucleotide sugar metabolism).

We applied the RRA algorithm to integrate and rank results from two datasets generated on different platforms. Using this approach, 26 significantly upregulated and 25 significantly downregulated genes were identified, with the top 20 upregulated and downregulated genes displayed in Figure 1A. Differential expression was defined as |logFC| > 0.585 with an adjusted *p* < 0.05. In addition, pooled differential expression analysis identified 26 upregulated and 19 downregulated genes, with the top 20 genes shown in Figure 1B. Taken together, a total of 40 *T. gondii* infection-related genes were ultimately determined (Figure 1C).

### 3.2. Biological Functions of the 40 T. gondii Infection-Related Genes

To further elucidate the biological functions of the 40 *T. gondii* infection-related genes, Gene Ontology (GO) and Kyoto Encyclopedia of Genes and Genomes (KEGG) enrichment analyses were performed. GO analysis indicated that these genes were mainly involved in cellular responses to external stimuli (including mechanical stimulus, progesterone, and ischemia), regulation of microRNA transcription and metabolism, and structural components related to ribosome biogenesis and cell adhesion. In terms of molecular function, significant enrichment was observed in protein binding activities, chromatin binding, and enzyme-related processes (Figure 1D,E). KEGG pathway analysis, based on unadjusted *p*-values, indicated that these genes were primarily associated with signaling and metabolic pathways, including cAMP signaling and carbohydrate metabolism (including galactose, fructose, mannose, and nucleotide sugar metabolism). Collectively, these findings suggest that *T. gondii* infection may influence host transcriptional regulation, stress responses, and metabolic processes in neuroepithelial cells (Figure 1F,G).

### 3.3. Construction and Validation of the Prognostic Model

**Figure 2:**
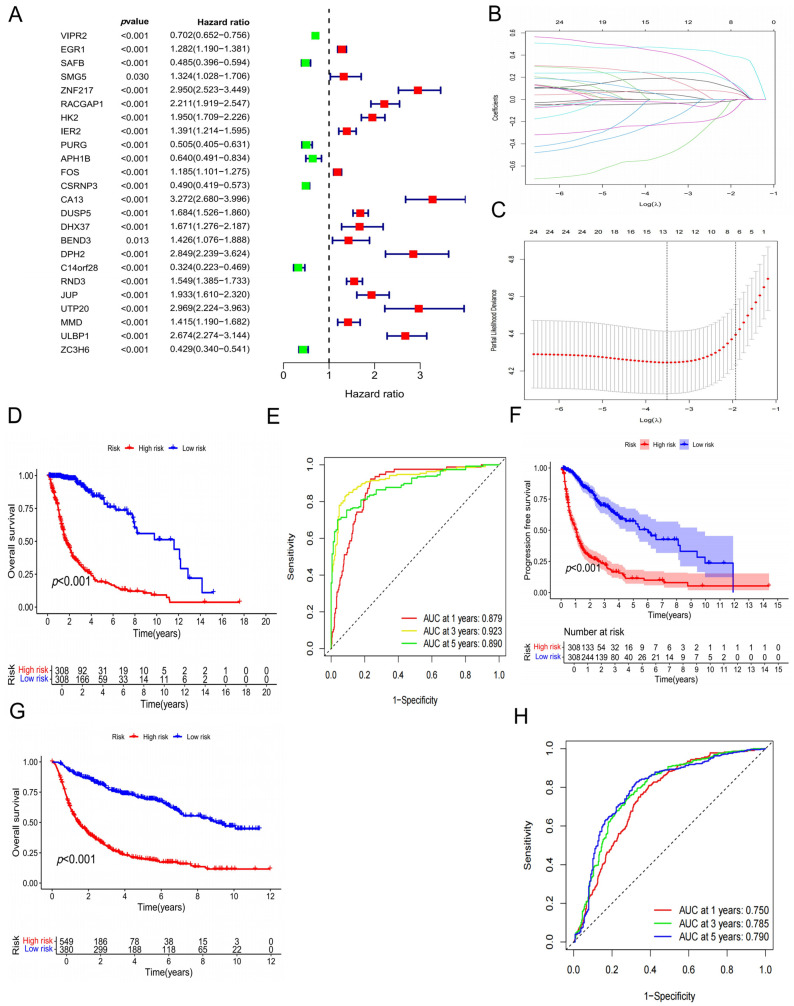
Construction and validation of the prognostic model based on *T. gondii* infection-related genes. (**A**) Forest plot of univariate Cox regression analysis identifying 24 *T. gondii* infection-related genes significantly associated with prognosis (*p* < 0.01). (**B**,**C**) LASSO regression analysis for feature selection. Coefficient profiles of candidate genes are shown in panel B, and the optimal lambda value determined by 10-fold cross-validation is presented in panel C. A total of 13 genes were retained for model construction. (**D**) Kaplan–Meier overall survival (OS) analysis in the training cohort, showing significantly worse survival in the high-risk group compared with the low-risk group (*p* < 0.001). (**E**) Time-dependent ROC curves in the training cohort, demonstrating strong predictive accuracy with AUCs of 0.879, 0.923, and 0.890 at 1, 3, and 5 years, respectively. (**F**) Kaplan–Meier progression-free survival (PFS) curves in the training cohort, indicating significantly shorter PFS in the high-risk group (*p* < 0.001). (**G**) Kaplan–Meier OS curves in the testing cohort, again showing significantly worse outcomes in the high-risk group (*p* < 0.001). (**H**) Time-dependent ROC curves in the testing cohort, with AUCs of 0.669, 0.603, and 0.645 at 1, 3, and 5 years, respectively.

In the training cohort, univariate Cox regression analysis identified 24 *T. gondii* infection-related genes that were significantly associated with prognosis (*p* < 0.01) (Figure 2A). LASSO regression further refined the candidate set, yielding 13 genes with their corresponding coefficients at the optimal lambda value (Figure 2B,C). These genes were then incorporated into a multivariate Cox regression model, resulting in the final prognostic signature. Based on their regression coefficients, we constructed the *T. gondii*-related Risk Score (TGRisk) (Supplementary Table S1).

Patients were stratified into high- and low-risk groups according to the median TGRisk. Scatter plots illustrated that increasing TGRisk scores were accompanied by shorter overall survival (OS) and higher mortality. Kaplan–Meier survival analysis indicated that patients in the high-risk group had significantly worse survival compared with those in the low-risk group (*p* < 0.001) (Figure 2D). In the training cohort, the predictive performance of the model was robust, with AUCs of 0.879, 0.923, and 0.890 at 1, 3, and 5 years, respectively (Figure 2E). Moreover, progression-free survival was also significantly shorter in the high-risk group (*p* < 0.001) (Figure 2F).

To further validate the robustness of the model, the same analysis was performed in the testing cohort. Consistent with the training set, patients in the low-risk group exhibited significantly longer OS (*p* < 0.001) (Figure 2G), with AUCs of 0.669, 0.603, and 0.645 at 1, 3, and 5 years, respectively (Figure 2H). Collectively, these findings demonstrate that the TGRisk model provides reliable prognostic predictive power across multiple independent cohorts.

Furthermore, to assess the prognostic significance of each candidate gene, the 13 genes incluTdjded in the TGRisk model were individually analyzed. For each gene, patients were divided into high- and low-expression groups according to the median expression level, and Kaplan–Meier survival curves were generated. The results indicated that most of these genes exhibited significant prognostic value, with high expression of risk-associated genes correlating with poorer OS, while protective genes showed the opposite trend (Figure S1).

### 3.4. Construction of the Nomogram and Decision Curve Analysis

**Figure 3:**
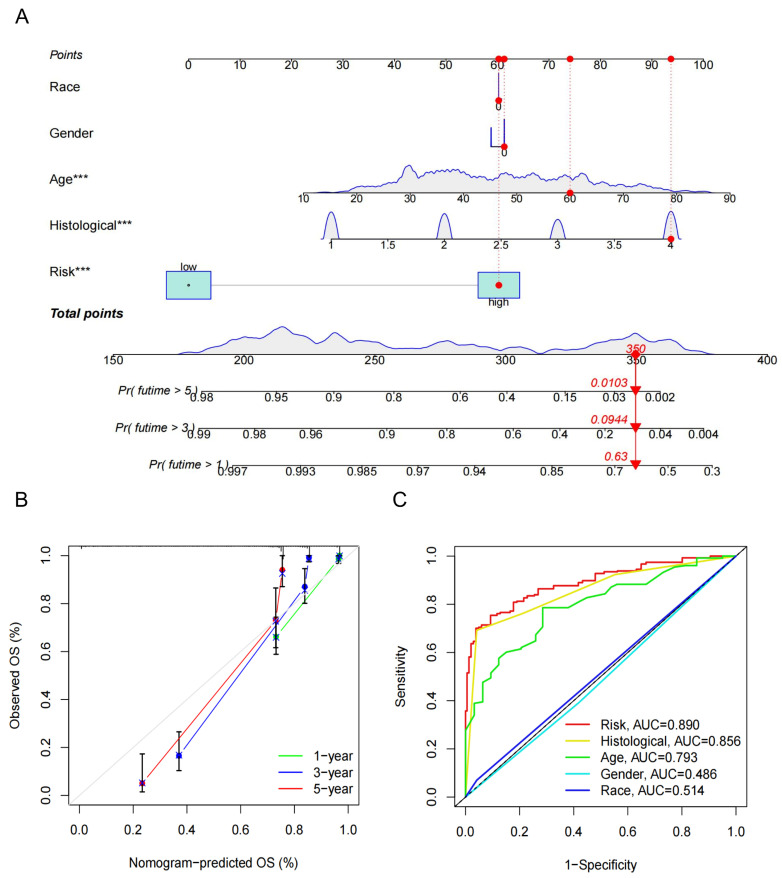
Construction and evaluation of the nomogram integrating TGRisk and clinical factors. (**A**) A prognostic nomogram incorporating TGRisk with clinical variables (race, gender, age, and histological subtype) for predicting 1-, 3-, and 5-year overall survival (OS) in glioma patients. To use the nomogram, an individual patient’s value is located on each variable axis, and a line is drawn upward to determine the points received for each variable. The sum of these points is located on the Total Points axis, and a line is drawn downward to the survival axes to determine the likelihood of survival. *** indicate *p* < 0.001. Red dots and arrows represent an example case calculation. (**B**) Calibration curves for 1-, 3-, and 5-year OS, showing strong concordance between nomogram-predicted and observed survival outcomes. (**C**) ROC curve analysis comparing predictive performance of the nomogram (risk score combined with clinical variables) and individual clinical factors, demonstrating superior accuracy of the TGRisk-based nomogram, with AUC values of 0.890, 0.856, and 0.793 for risk score, histology, and age, respectively.

We constructed a nomogram integrating clinical factors with TGRisk to predict 1-, 3-, and 5-year survival probabilities in glioma patients (Figure 3A). Calibration curves indicated strong agreement between predicted and observed outcomes (Figure 3B). In addition, the AUC results indicated that the nomogram achieved superior clinical net benefit in prognostic prediction at 1, 3, and 5 years (Figure 3C). These findings suggest that the TGRisk-based nomogram may serve as an effective tool for prognostic prediction in clinical practice.

### 3.5. Functional Enrichment Analysis

**Figure 4:**
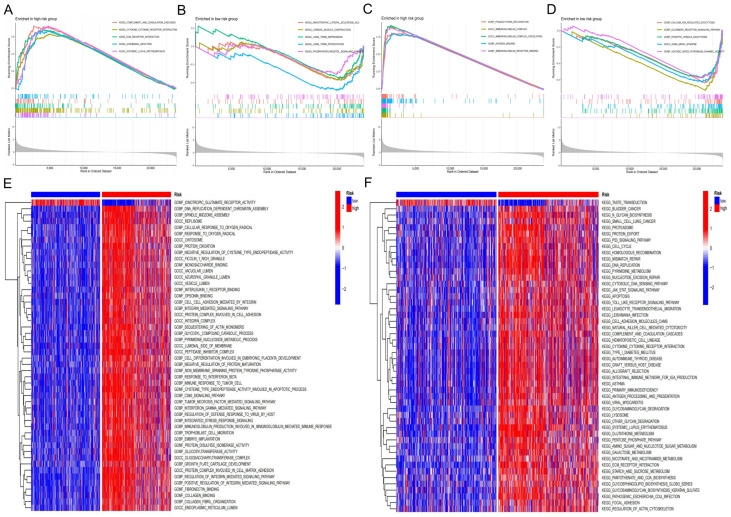
Functional enrichment analysis between high- and low-risk groups. (**A**–**D**) Gene Set Enrichment Analysis (GSEA) comparing high- and low-risk glioma groups. The high-risk group was significantly enriched in immune and inflammatory pathways, including complement and coagulation cascades, cytokine–cytokine receptor interaction, ECM–receptor interaction, and systemic lupus erythematosus (**A**,**C**). In contrast, the low-risk group showed enrichment in neuronal and metabolic pathways such as amyotrophic lateral sclerosis (ALS), cardiac muscle contraction, phosphatidylinositol signaling, synaptic vesicle exocytosis, and glutamate receptor signaling (**B**,**D**). (**E**,**F**) Gene Set Variation Analysis (GSVA) heatmaps of representative GO terms (**E**) and KEGG pathways (**F**). The high-risk group was enriched in processes related to cell cycle regulation, DNA replication, oxidative stress responses, immune activation, and extracellular matrix organization, whereas the low-risk group was predominantly enriched in neuronal receptor signaling processes, including taste transduction and ionotropic glutamate receptor activity.

Functional enrichment analysis using both GSEA and GSVA indicated distinct biological characteristics between the two risk groups. In the training cohort, GSEA showed that the high-risk group was mainly enriched in immune-related and inflammatory pathways, including complement and coagulation cascades, cytokine–cytokine receptor interaction, ECM–receptor interaction, Leishmania infection, systemic lupus erythematosus, phagocytosis recognition, immunoglobulin complex, and antigen receptor binding, suggesting an activated immune and inflammatory state. In contrast, the low-risk group was enriched in neuronal and metabolic pathways, such as amyotrophic lateral sclerosis (ALS), cardiac muscle contraction, phosphatidylinositol signaling, synaptic vesicle exocytosis, and glutamate receptor signaling (Figure 4A–D).

Consistently, GSVA further identified that the high-risk group was enriched in cell cycle regulation, DNA replication, oxidative stress responses, immune activation, and extracellular matrix organization, whereas the low-risk group was predominantly enriched in receptor-mediated neuronal signaling processes, including taste transduction and ionotropic glutamate receptor activity (Figure 4E,F).

### 3.6. Differential Immune Landscape Between High- and Low-Risk Groups

**Figure 5:**
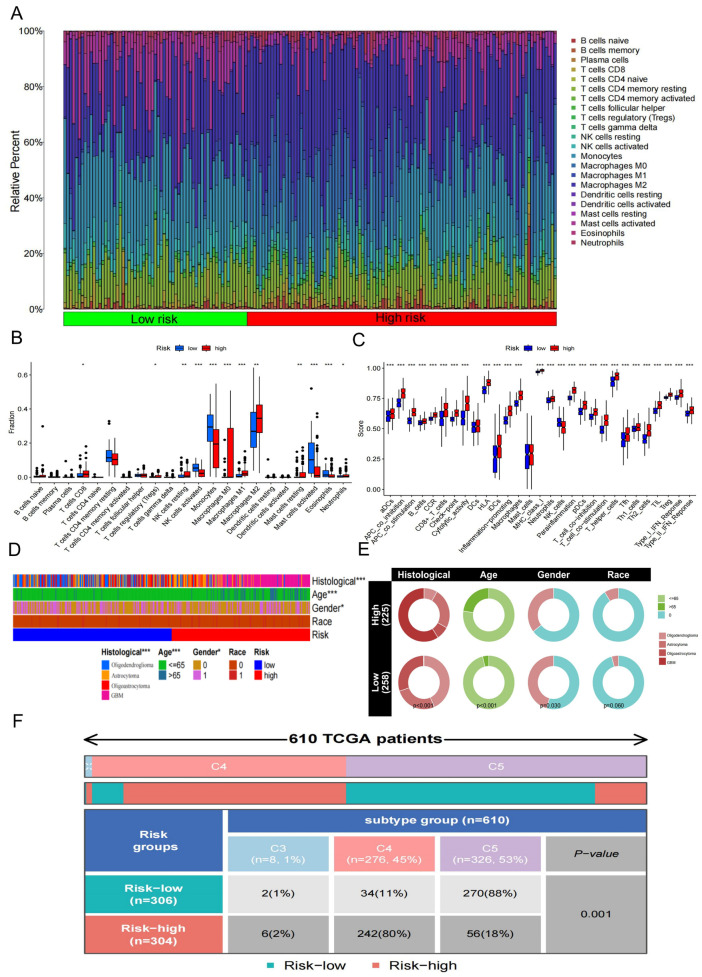
Differential immune landscape and clinicopathological associations between high- and low-risk groups. (**A**) Stacked bar plots showing the relative proportions of 22 tumor-infiltrating immune cell types estimated by CIBERSORT in glioma patients, stratified by high- and low-risk groups. (**B**) Box plots of immune infiltration comparing high- and low-risk groups. The high-risk group exhibited significantly higher proportions of CD8^+^ T cells, regulatory T cells (Tregs), resting NK cells, macrophages (M0, M1, M2), resting mast cells, and neutrophils, whereas the low-risk group showed enrichment of activated NK cells, activated mast cells, and eosinophils. (**C**) ssGSEA-based functional enrichment of immune-related processes. The high-risk group was enriched in APC co-inhibition/co-stimulation, B cell and CD8^+^ T cell activation, checkpoint signaling, HLA expression, cytolytic activity, MHC class I presentation, neutrophil and macrophage activation, parainflammation, Th1/Th2/Tfh responses, tumor-infiltrating lymphocytes, and interferon responses, while the low-risk group was selectively enriched in NK cell-related functions. (**D**) Clinical heatmap illustrating the associations of risk groups with histological subtype, age, gender, and race. (**E**) Circular composition plots showing significantly different distributions of histological subtype and age across risk groups. High-risk patients had a greater proportion of GBM and were more likely to be older (>65 years), while oligodendrogliomas were predominant in the low-risk group. (**F**) Immune subtype distribution in high- and low-risk groups. High-risk patients were primarily enriched in the C4 immune subtype, whereas the low-risk group clustered mainly in the C5 subtype (*p* = 0.001). * *p* < 0.05; ** *p* < 0.01; *** *p* < 0.001.

The tumor microenvironment consists of multiple components, including cancer-associated fibroblasts, immune cells, extracellular matrix, various growth factors, inflammatory mediators, unique physicochemical properties, and tumor cells themselves. It exerts a profound influence on the diagnosis, survival, and treatment sensitivity of malignant tumors. The proportions of tumor-infiltrating immune cells in each patient from the high- and low-risk groups are shown in Figure 5A. Immune infiltration analysis suggested distinct immune cell patterns between the two risk groups. In the high-risk group, the proportions of multiple immunosuppressive and pro-tumor immune cells were significantly elevated, including CD8^+^ T cells, regulatory T cells (Tregs), resting NK cells, macrophages (M0, M1, M2), resting mast cells, and neutrophils, suggesting an immune-activated yet dysregulated microenvironment. In contrast, the low-risk group exhibited significantly higher levels of activated NK cells, activated mast cells, and eosinophils, indicating a more effective anti-tumor immune response (Figure 5B).

Consistently, functional analysis based on ssGSEA identified that the high-risk group was enriched in a wide range of immune-related processes, such as antigen-presenting cell (APC) co-inhibition/co-stimulation, B cell and CD8^+^ T cell activation, checkpoint signaling, HLA expression, cytolytic activity, MHC class I presentation, neutrophil and macrophage activation, parainflammation, T helper cell (Th1, Th2, Tfh) responses, tumor-infiltrating lymphocytes (TILs), as well as type I and II interferon responses. Conversely, the low-risk group showed a selective enrichment in NK cell-related functions, highlighting differential immune activation patterns between the two groups (Figure 5C).

### 3.7. Association of Risk Score with Clinicopathological Features and Immune Subtypes

The results showed that the risk score was closely associated with multiple clinicopathological characteristics. Clinical heatmap analysis indicated significant differences in histological subtype, age, and sex between the high- and low-risk groups (Figure 5D). Circular composition plots further suggested distinct distributions of histological type and age across risk groups, with a higher proportion of GBM cases observed in the high-risk group, whereas oligodendrogliomas predominated in the low-risk group. In addition, patients in the high-risk group were more likely to be older (>65 years) (Figure 5E). Moreover, immune subtype analysis showed a significant association between risk groups and immune subtype distribution (*p* = 0.001), with high-risk patients predominantly enriched in the C4 subtype, while the low-risk group was mainly clustered in the C5 subtype (Figure 5F). Collectively, these findings suggest that the risk model not only reflects prognostic differences but is also strongly correlated with clinicopathological features such as histological classification, age, and immune subtype.

### 3.8. Drug Sensitivity Analysis

**Figure 6:**
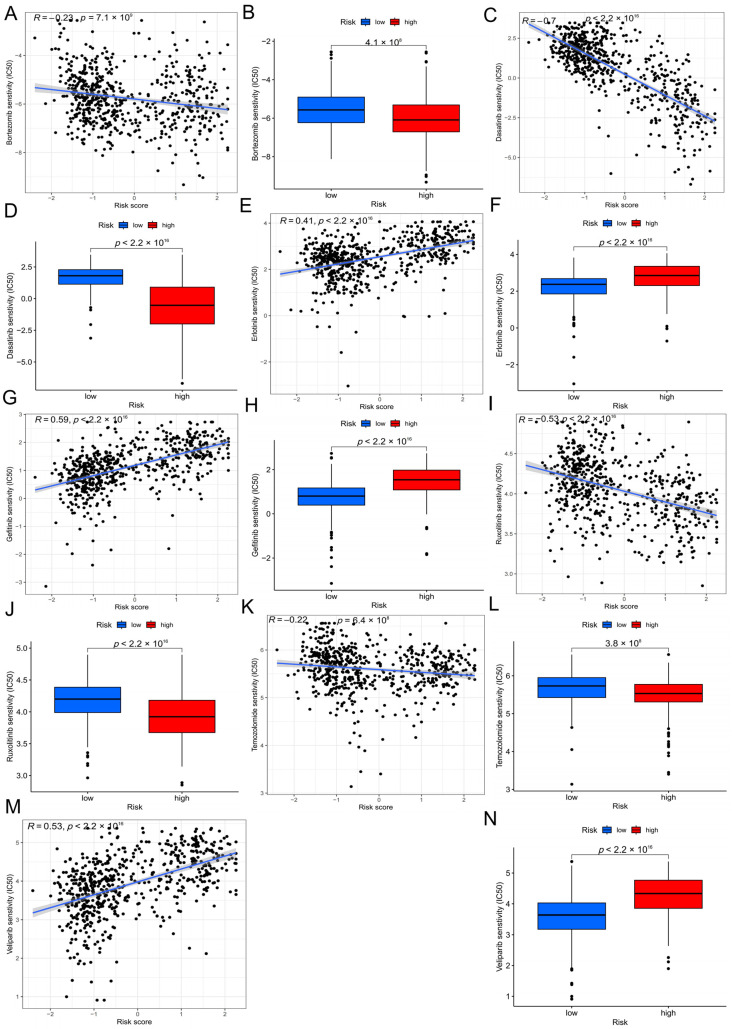
Drug sensitivity analysis between high- and low-risk groups. (**A**,**B**) Bortezomib: the low-risk group exhibited significantly higher sensitivity (lower IC50). (**C**,**D**) Dasatinib: the high-risk group showed significantly greater sensitivity (lower IC50). (**E**,**F**) Erlotinib: the high-risk group displayed relative resistance, with higher IC50 values. (**G**,**H**) Gefitinib: consistent with erlotinib, the high-risk group exhibited higher IC50 values, suggesting resistance. (**I**,**J**) Ruxolitinib: the high-risk group had significantly lower IC50 values, indicating greater sensitivity. (**K**,**L**) Temozolomide: the low-risk group showed enhanced sensitivity (lower IC50), supporting its role as a standard therapy in glioma. (**M**,**N**) Veliparib: the high-risk group exhibited higher IC50 values, indicating relative resistance.

We further evaluated the potential therapeutic response between high- and low-risk groups based on drug sensitivity prediction. The results suggested that patients in the low-risk group exhibited significantly higher sensitivity to temozolomide (the standard chemotherapeutic agent for glioma) and bortezomib, suggesting a potential benefit from these treatments. Conversely, the high-risk group showed significantly lower IC50 values for dasatinib and ruxolitinib, indicating greater sensitivity to these targeted agents. In contrast, erlotinib, gefitinib, and veliparib showed higher IC50 values in the high-risk group, implying relative resistance. Collectively, these findings indicate that the risk signature not only stratifies patient prognosis but may also provide guidance for personalized therapeutic strategies in glioma (Figure 6).

### 3.9. Validation of the TGRisk Signature via RT-qPCR in Glioma Cell Lines

To evaluate the expression profiles of the key genes within the TGRisk model, we performed RT-qPCR across a biological gradient consisting of normal HA, low-risk U251 cells, and high-risk, TMZ-resistant T98G cells. The experimental results were highly concordant with the prognostic directions identified in our bioinformatic analysis.

**Figure 7:**
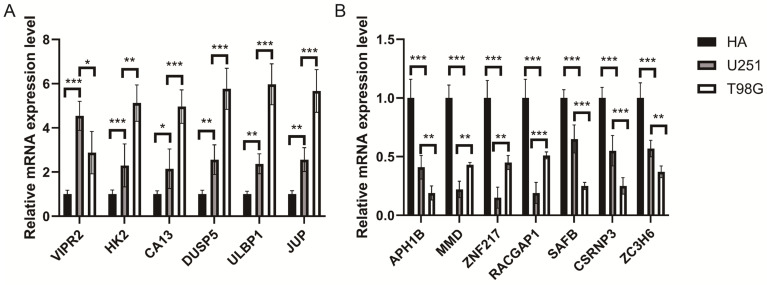
Detection of target genes’ mRNA levels in human glioma cells, U251 cells, and T98G cells by RT-qPCR. (**A**) Quantitative RT-PCR validation of VIPR2, HK2, CA13, DUSP5, ULBP1, and JUP mRNA levels in HA, U251, and T98G cells. (**B**) Quantitative RT-PCR validation of APH1B, MMD, ZNF217, RACGAP1, SAFB, CSRNP3, and ZC3H6 mRNA levels. Expression levels were normalized to GAPDH and are presented relative to the calibrator sample (HA). Data are shown as mean ± SD. * *p* < 0.05, ** *p* < 0.01, *** *p* < 0.001.

Specifically, the eight risk-associated genes (HK2, CA13, ZNF217, RACGAP1, DUSP5, MMD, ULBP1 and JUP) exhibited a significant increase in mRNA expression from the T98G group compared to that in the U251 group. Conversely, the five protective genes (VIPR2, SAFB, APH1B, CSRNP3 and ZC3H6) were markedly down-regulated in T98G group compared to U251 group (Figure 7). This characteristic loss of protective transcriptional programs in the high-risk group supports the premise that such molecular dysregulation is associated with advanced histological grade, older age, and diminished sensitivity to standard chemotherapy, such as temozolomide.

## 4. Discussion

Gliomas remain among the most lethal primary brain tumors, characterized by extensive heterogeneity and limited therapeutic options. Despite advances in molecular classification and limited therapeutic options, reliable biomarkers that integrate prognosis with potential therapeutic implications are still lacking. In this study, we explored the prognostic value of *T. gondii* infection-related genes. Our rationale was not the generic claim that cancer and infection share pathways, but rather a host transcriptional program may index a distinct immune–metabolic state in brain tumors and thereby offer insights beyond conventional pathway enrichment. By integrating multi-cohort transcriptomic datasets, we identified a robust prognostic signature (TGRisk) that stratifies patients into subgroups with different outcomes, immune characteristics, and drug sensitivity patterns.

Through robust rank aggregation, we identified 40 differentially expressed genes related to *T. gondii* infection. Functional enrichment analyses suggested that these genes participate in cellular responses to stress, microRNA transcriptional regulation, ribosome biogenesis, and metabolic processes such as carbohydrate and nucleotide metabolism. Such pathways reflect the parasite’s known ability to reprogram host transcription and metabolism [31,32]. Recent experimental evidence supports this crosstalk [15,16]. Rather than treating this as mere “overlap,” we interpret the combined stress–translation–metabolism module as a coherent malignant fitness program in glioma, a concrete biological reason why a parasite-linked signature may carry information beyond the broad, non-specific inflammatory terms commonly produced by generic pathway analyses.

Based on Cox and LASSO regression, we developed the TGRisk model composed of 13 genes. This signature exhibited strong prognostic power across training, testing, and validation cohorts, with significantly worse survival observed in the high-risk group. Importantly, the predictive accuracy of TGRisk was consistently high, underscoring its robustness. Unlike prior glioma prognostic signatures focused on immune checkpoints [33], DNA repair [34], or metabolism [35], our model is infection-related, thus introducing a novel biological perspective. The value of this perspective lies in interpretability: it connects prognosis to an immune–metabolic remodeling axis that can be interrogated experimentally and potentially leveraged therapeutically, rather than serving as a purely statistical classifier. We further integrated TGRisk with conventional clinical variables in a nomogram, which showed good calibration and decision curve performance, supporting potential clinical utility. This aligns with the growing emphasis on combining molecular signatures with clinicopathological variables for personalized oncology [36].

Functional enrichment analyses further illustrated distinct biological landscapes between risk groups. GSEA showed enrichment of immune and inflammatory pathways in the high-risk group, including complement cascades, cytokine–cytokine receptor interactions, and phagocytosis processes, while the low-risk group was enriched in neuronal signaling pathways such as glutamate receptor signaling. We interpret this as a state shift from a more neuronal-like or differentiated profile in the low-risk group to an injury-like, inflammatory, and proliferative profile in the high-risk group, a conceptual framework that provides a meaningful basis for the observed survival divergence. Immune infiltration supported this interpretation: high-risk tumors exhibited increased CD8^+^ T cells together with Tregs, macrophage subsets, and neutrophils, and ssGSEA suggested enrichment of checkpoint signaling, HLA expression, interferon pathways, and broader immune activity; meanwhile, low-risk tumors showed higher activated NK-related features [37,38]. This “inflamed-but-dysfunctional” ecosystem offers a biological explanation for why immune activation signatures may coincide with poor outcomes, consistent with the association of high TGRisk with GBM histology, older age, and C4 immune subtype, and low risk with oligodendroglioma and C5 [39].

Drug sensitivity prediction further suggested translational relevance. While these findings are derived from the pRRophetic computational framework and currently represent a lower level of clinical evidence compared to prospective trials, they offer a data-driven roadmap for drug repositioning. Low-risk patients were predicted to be more sensitive to temozolomide and bortezomib, whereas high-risk patients were predicted to respond better to dasatinib and ruxolitinib and to show relative resistance to EGFR and PARP inhibitors [40,41]. Within our framework, we speculate that TGRisk-low tumors may remain more vulnerable to cytotoxic or proteostasis stress, while TGRisk-high tumors, characterized by inflammatory remodeling and suppressive immune circuits, may depend more on kinase and cytokine or JAK–STAT–linked signaling vulnerabilities. This yields testable hypotheses. TGRisk-high tumors should exhibit functional T cell exhaustion and myeloid or Treg-mediated suppression despite higher CD8^+^ abundance. Perturbing the inferred signaling dependencies (including JAK/SRC-axis) should partially rewire immune dysfunction and enhance response to standard therapy in high-risk models. Furthermore, given that the upstream program emphasizes ribosome biogenesis and metabolism, perturbing key nodes within the 13-gene set should preferentially impair stress tolerance and proliferation in TGRisk-high glioma cells.

Finally, several limitations and conceptual nuances warrant careful consideration. A critical conceptual limitation warrants explicit consideration; the regulatory mechanisms underlying this association may differ between acute infection and the glioma tumor microenvironment [42]. In the acute infection setting, host immune responses are primarily geared toward pathogen clearance [43]. This distinction suggests that the transcriptional program captured by TGRisk reflects a sustained, context-dependent rewiring of host pathways rather than a direct recapitulation of acute infection responses [17,42]. Moreover, we adopted the label “*T. gondii* infection-related genes” based on transcriptomic signatures originally characterized in acute infection models. While *T. gondii* is a neurotropic parasite and epidemiological evidence links seropositivity to brain tumors [13,14], our signature reflects a host transcriptional program responsive to infection-related stimuli rather than direct evidence of parasite presence [42]. Consequently, the “infection-related” designation should be interpreted as a shared host-response program, potentially activated by diverse stress signals within the tumor, rather than as proof of active *T. gondii* infection [44,45]. Second, the observed parasite-related effects are based on bioinformatics mapping and remain indirect. Third, although we validated gene expression levels by RT-qPCR, our study lacks in-depth functional experiments. Future research using gene knockdown or animal models is needed to clarify the exact mechanisms by which these 13 genes contribute to glioma progression. Fourth, the drug sensitivity predictions are computationally derived. Their clinical relevance requires validation through pharmacodynamic studies and prospective trials. Finally, future studies should incorporate serological or pathological evidence to determine whether the transcriptional program captured by TGRisk involves similar molecular pathways [14,46,47]. On this basis, efforts should focus on glioma cohorts with confirmed *T. gondii* seropositivity and integrate emerging technologies such as spatial transcriptomics to further reveal the actual interactions between pathogen components and the tumor microenvironment.

## 5. Conclusions

This study presents a novel *T. gondii* infection-related gene signature, termed TGRisk, which robustly stratifies glioma patients by prognosis, immune features, and therapeutic vulnerabilities. The findings illustrate cross-kingdom regulation at multiple levels. In this way, infection biology and tumor biology, traditionally viewed as distinct fields, become interconnected. Additionally, TGRisk holds potential as a prognostic and precision therapeutic biomarker for gliomas, representing a direct application of the cross-kingdom regulation concept.

### 5.1. Limitations

Several limitations warrant acknowledgment. The regulatory mechanisms of *T. gondii* infection-related genes may differ significantly between the glioma microenvironment and the acute infection setting. This study was retrospective in design and relied exclusively on public transcriptomic datasets, without incorporating glioma patients with confirmed *T. gondii* infection. The causal relationship between the infection-associated transcriptional program captured by TGRisk and glioma biology therefore remains to be established. In addition, the biological functions of candidate genes have yet to be experimentally defined; their roles in glioma are currently speculative. Furthermore, the drug sensitivity predictions—particularly those involving dasatinib and ruxolitinib—are derived solely from computational estimates. Whether these agents exert the predicted effects in vivo requires direct experimental validation.

### 5.2. Future Perspectives

Addressing these limitations, future research should prioritize multi-level experimental validation. Ideally, such validation should incorporate serological or pathological evidence of *T. gondii* infection in clinical samples. Complementary mechanistic studies—such as knockdown or overexpression experiments targeting the 13 identified genes—would also help substantiate the functional relevance of this signature. Mechanistic studies using both in vitro and in vivo models are also essential to clarify how infection-related genes contribute to glioma biology. From a therapeutic perspective, the predicted vulnerabilities to dasatinib and ruxolitinib in high-risk gliomas warrant systematic evaluation. These agents could be tested alone or in combination with immunotherapies, potentially opening new treatment avenues for this challenging malignancy.

## Data Availability

The datasets supporting the conclusions of this study are publicly available. TCGA data can be accessed via https://portal.gdc.cancer.gov (accessed on 1 December 2025, CGGA data via http://www.cgga.org.cn/ (accessed on 1 December 2025), and GEO data under accession GSE22986 (https://www.ncbi.nlm.nih.gov/geo/query/acc.cgi?acc=GSE22986 (accessed on 1 December 2025).

## Supplementary Materials

The following supporting information can be downloaded at: https://www.mdpi.com/article/10.3390/biology15080633/s1, Figure S1: Prognostic significance of the 13 genes included in the TGRisk model; Table S1: The 13-gene *T. gondii*-related prognostic signature (TGRisk) and corresponding regression coefficients at the optimal lambda value derived from LASSO regression. Table S2: Specific databases, patient numbers, and cohort roles (training vs. validation) in glioma cohorts used for model construction and validation.

## Abbreviations

The following abbreviations are used in this manuscript:

GBM	Glioblastoma
GEO	Gene Expression Omnibus
DE	Differential expression
DEGs	Differentially expressed genes
RRA	Robust Rank Aggregation
GO	Gene Ontology
KEGG	Genes and Genomes
BP	Biological processes
MF	Molecular functions
GSVA	Gene set variation analysis
GSEA	Gene set enrichment analysis
