# Supplementary material for: Development and Validation of a Toxoplasma Infection-Associated Risk Model for Prognostic Stratification and Treatment Guidance in Glioma
Source: Biology (Basel). 2026 Apr 17;15(8):633. doi: 10.3390/biology15080633 (PMC13113236; doi:10.3390/biology15080633)
Supplement: Supplementary file 1 [file biology-15-00633-s001.zip › Supplementary Figure S1.pdf]

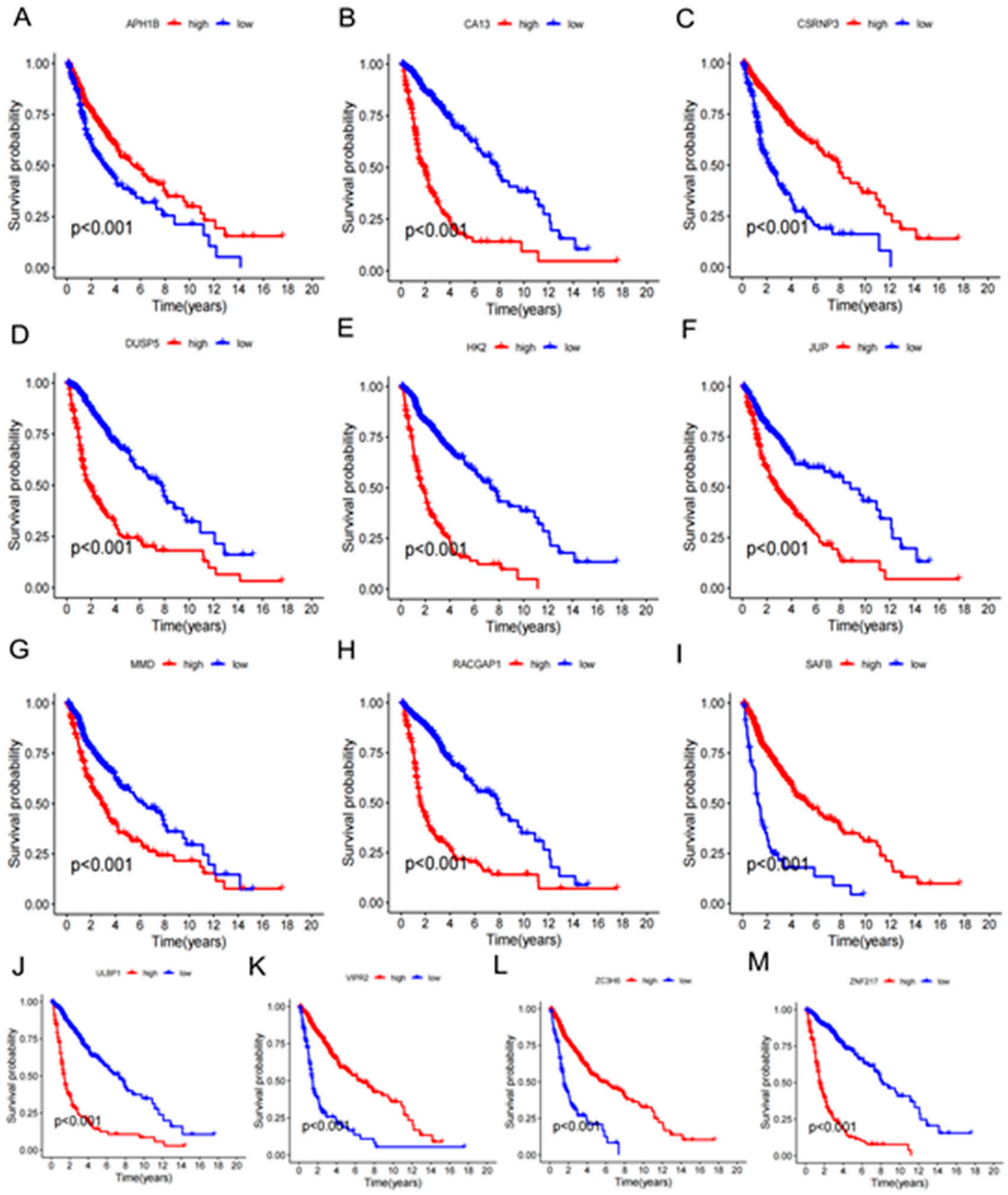

Figure S1. Prognostic significance of the 13 genes included in the TGRisk model.
(A–M) High expression of risk-associated genes, including APH1B, CA13, CSRNP3, DUSP5, HK2, JUP, MMD, RACGAP1, SAFB, ULBP1, and ZNF217, was significantly correlated with poorer overall survival (OS). In contrast, high expression of protective genes, such as VIPR2 and ZC3H6, was associated with improved OS. Log-rank test P-values are indicated in each panel. Collectively, these results support the independent prognostic value of the genes constituting the TGRisk model.
