# Supplementary material for: Development and Validation of a Toxoplasma Infection-Associated Risk Model for Prognostic Stratification and Treatment Guidance in Glioma
Source: Biology (Basel). 2026 Apr 17;15(8):633. doi: 10.3390/biology15080633 (PMC13113236; doi:10.3390/biology15080633)
Supplement: Supplementary file 1 [file biology-15-00633-s001.zip › Supplementary Table S1.pdf]

Supplementary Table S1. The 13-gene *T. gondii*–related prognostic signature (TGRisk) and corresponding regression coefficients at the optimal lambda value derived from LASSO regression.

**Gene**	VIPR2	SAFB	ZNF217	RACGAP1	HK2	APH1B	CSRNP3
**Coef**	-0.037603606	-0.496976722	0.464886773	0.422118536	0.047754051	-0.188679399	-0.222037471

**Gene**	CA13	DUSP5	JUP	MMD	ULBP1	ZC3H6
**Coef**	0.192050383	0.083636579	0.194427514	0.024893661	0.24408394	-0.026703144
