# Supplementary material for: Development and Validation of a Toxoplasma Infection-Associated Risk Model for Prognostic Stratification and Treatment Guidance in Glioma
Source: Biology (Basel). 2026 Apr 17;15(8):633. doi: 10.3390/biology15080633 (PMC13113236; doi:10.3390/biology15080633)
Supplement: Supplementary file 1 [file biology-15-00633-s001.zip › Supplementary Table S2.pdf]

Supplementary Table S2. Specific databases, patient numbers, and cohort roles (training vs. validation) in glioma cohorts used for model construction and validation.

Database	Cohort Role	Number of Samples (N)	Clinical Data Used
TCGA (The Cancer Genome Atlas)	Training & Discovery	616	RNA-seq, Survival time, Survival status
CGGA (Chinese Glioma Genome Atlas)	External Validation	929	RNA-seq, Survival time, Survival status
